# High prevalence of thiamine (vitamin B1) deficiency in early childhood among a nationally representative sample of Cambodian women of childbearing age and their children

**DOI:** 10.1371/journal.pntd.0005814

**Published:** 2017-09-05

**Authors:** Kyly C. Whitfield, Geoffry Smith, Chhoun Chamnan, Crystal D. Karakochuk, Prak Sophonneary, Khov Kuong, Marjoleine Amma Dijkhuizen, Rathavuth Hong, Jacques Berger, Tim J. Green, Frank Tammo Wieringa

**Affiliations:** 1 Department of Applied Human Nutrition, Mount Saint Vincent University, Halifax, Nova Scotia, Canada; 2 International Life Sciences Institute (Southeast Asia Region), Singapore; 3 Essential Micronutrients Foundation, Singapore; 4 Department of Post-Harvest Technologies and Quality Control, Fisheries Administration, Ministry of Agriculture, Forestry and Fisheries, Phnom Penh, Cambodia; 5 Food, Nutrition, and Health, University of British Columbia, Vancouver, British Columbia, Canada; 6 National Nutrition Programme, Maternal and Child Health Centre, Phnom Penh, Cambodia; 7 Nutrition, Exercise and Sports, Copenhagen University, Copenhagen, Denmark; 8 ICF International, Rockville, Maryland, United States of America; 9 Institute of Research for Development (IRD), UMR-204, IRD-UM-SupAgro, Montpellier, France; 10 Healthy Mothers, Babies and Children Theme, South Australian Health and Medical Research Institute, Adelaide, South Australia, Australia; 11 Discipline of Paediatrics and Reproductive Health, University of Adelaide, Adelaide, South Australia, Australia; University of Washington, UNITED STATES

## Abstract

**Background:**

Thiamine deficiency is thought to be an issue in Cambodia and throughout Southeast Asia due to frequent clinical reports of infantile beriberi. However the extent of this public health issue is currently unknown due to a lack of population-representative data. Therefore we assessed the thiamine status (measured as erythrocyte thiamine diphosphate concentrations; eThDP) among a representative sample of Cambodian women of childbearing age (15–49 y) and their young children (6–69 mo).

**Methodology/Principle findings:**

Samples for this cross-sectional analysis were collected as part of a national micronutrient survey linked to the Cambodian Demographic and Health Survey (CDHS) 2014. One-sixth of households taking part in the CDHS were randomly selected and re-visited for additional blood sampling for eThDP analysis (719 women and 761 children). Thiamine status was assessed using different cut-offs from literature.

Women were mean (SD) 30 (6) y, and children (46% girls) were 41 (17) mo. Women had lower mean (95% CI) eThDP of 150 nmol/L (146–153) compared to children, 174 nmol/L (171–179; *P* < 0.001). Using the most conservative cut-off of eThDP < 120 nmol/L, 27% of mothers and 15% of children were thiamine deficient, however prevalence rates of deficiency were as high as 78% for mothers and 58% for children using a cut-off of < 180 nmol/L. Thiamine deficiency was especially prevalent among infants aged 6–12 mo: 38% were deficient using the most conservative cut-off (< 120 nmol/L).

**Conclusions/Significance:**

There is a lack of consensus on thiamine status cut-offs; more research is required to set clinically meaningful cut-offs. Despite this, there is strong evidence of suboptimal thiamine status among Cambodian mothers and their children, with infants <12 mo at the highest risk. Based on eThDP from this nationally-representative sample, immediate action is required to address thiamine deficiency in Cambodia, and likely throughout Southeast Asia.

## Author summary

Thiamine is an often-overlooked micronutrient of concern in Cambodia and throughout Southeast Asia, where reports of beriberi are not uncommon due to a diet of thiamine-poor, white, polished rice. Thiamine plays a critical role in cellular energy generation, and also modulates neuronal and neuromuscular transmissions. Thiamine deficiency can progress to beriberi, which can be fatal. Although several recent studies have investigated thiamine status and/or beriberi in the region, this is the first nationally-representative biochemical thiamine data from any country in Southeast Asia. Unfortunately, there is a lack of clinically meaningful cut-offs to interpret these data. Upwards of 10 different cut-offs exist, but many are simply the lower bounds of a reference range, and therefore do not align with clinical symptoms of beriberi. Using the most conservative cut-off from the literature, 27% of mothers and 15% of children were thiamine deficient. More research is required to develop more useful, clinically meaningful thiamine status cut-offs. In addition, given the distinctive peak in Cambodian infant mortality data suggestive of infantile beriberi, immediate action is required to improve the thiamine status in Cambodia, and likely in other countries in Southeast Asia.

## Introduction

Beriberi is a ‘forgotten disease’ [1–4] that remains a public health issue in Southeast Asia despite near eradication elsewhere [5–7]. Beriberi is caused by thiamine (vitamin B_1_) deficiency, and is most serious in infants due to the rapid growth and development that occurs during this time, and the relatively high thiamine needs compared to body size [4,8]. Breast milk thiamine concentrations reflect maternal dietary thiamine intake [9]. As such, poor maternal thiamine status during pregnancy and lactation puts infants at risk of developing beriberi [9–11], which can lead to death in hours of clinical presentation if not recognized or left untreated [6]. While infantile beriberi is the most serious outcome of thiamine deficiency, marginal thiamine status in the wider population causes fatigue, apathy, anorexia, and dizziness [12], and with these the potential for decreased school performance and/or economic output. In addition, Israeli children who consumed thiamine-deficient infant formula in infancy, but did not develop beriberi, exhibited retarded neurological, cognitive, and cardiological development at age 5–7 y [13], highlighting the importance of thiamine sufficiency in early life.

Beriberi remains a problem in Southeast Asia, in part, because non-parboiled [14], unfortified white rice is the dietary staple [8,12]. In Cambodia, white rice makes up an estimated 60% of daily dietary energy [15]. Although rice contains thiamine [8], it is found only in the outer husk and bran, the vast majority of which is removed during the milling process [14]. In most rice-consuming cultures, polished white rice is preferred [14,16] for several reasons: organoleptic qualities, white rice as a status symbol [12], and because removal of the lipid-rich outer bran increases shelf-life [14]. In Cambodia brown rice is a cultural dietary taboo; people were forced to eat brown rice during the Khmer Rouge regime [17].

**Fig 1:**
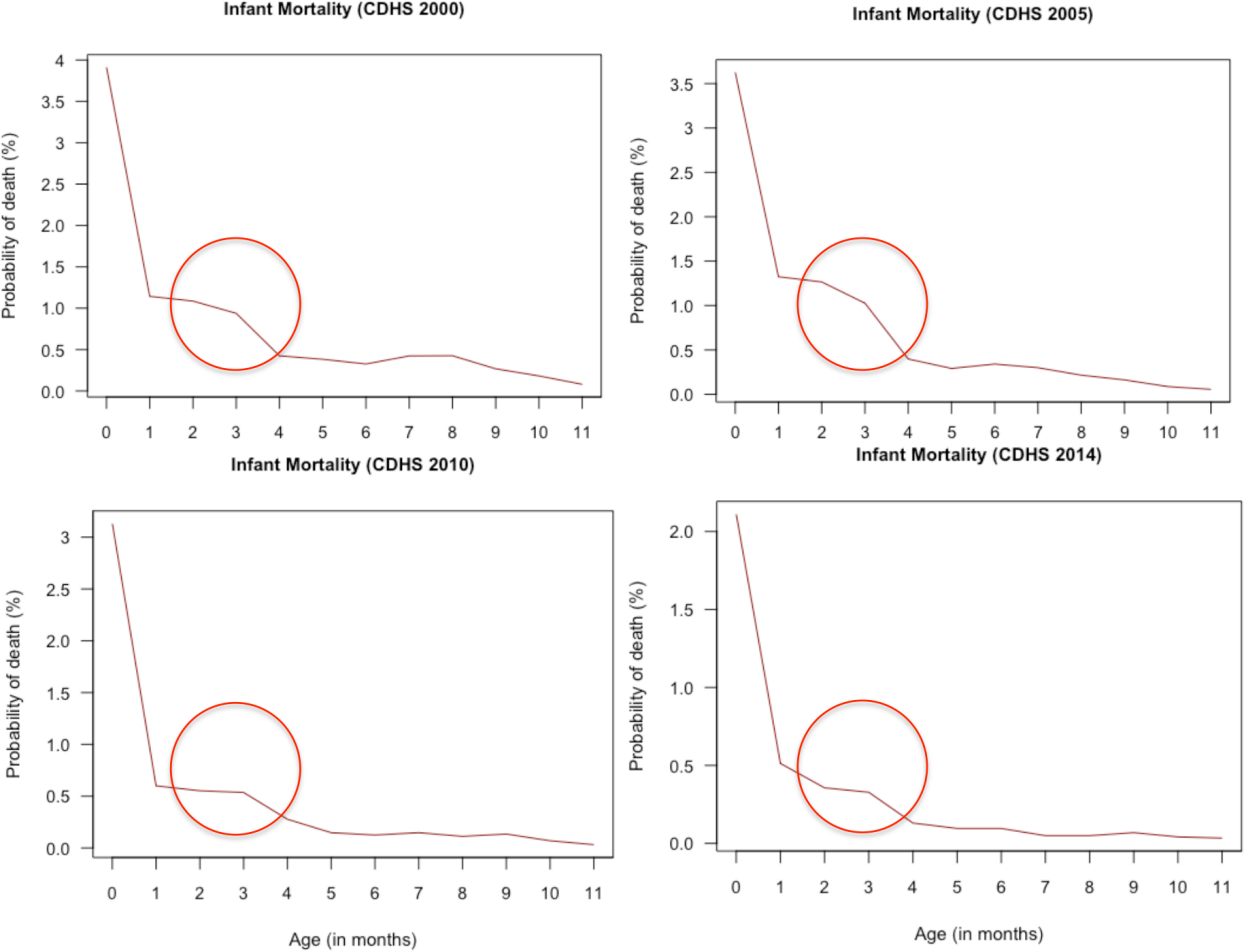
Infant mortality probability obtained from the Cambodian Demographic and Health Surveys between 2000 and 2014. The red circle highlights a peak in mortality around 3 months of age.

Although there are several reports of infantile beriberi in Cambodia [5,18,19], there is a lack of accurate prevalence data. The World Health Organization has suggested that in the absence of reliable information on the prevalence of beriberi or on biochemical markers of thiamine status, infant mortality curves could be indicative of thiamine deficiency being a health concern, with a peak in infant mortality around 3–4 months of age being suggestive of a high prevalence of beriberi [20]. We analyzed infant mortality data from the Cambodian Demographic Health Surveys (CDHS) 2000, 2005, 2010 and 2014 and found indeed a peak in mortality around 3 months of age (**Fig 1**).

Thiamine status has traditionally been assessed using a functional indicator, erythrocyte transketolase activity coefficient [21,22], however, this method has several shortcomings including the inactivation of transketolase during sample processing and storage, poor inter-assay precision [23], and a tendency of this assay to underreport deficiency among chronically deficient individuals [24]. More recently, the biochemical assessment of the biologically active form of vitamin B_1_, thiamine diphosphate, in whole blood or in erythrocytes has been advocated [2]. Erythrocyte thiamine diphosphate concentrations (eThDP) measured by high performance liquid chromatography (HPLC) overcomes several downfalls of the functional assay, and correlates well with erythrocyte transketolase activity coefficient [23]. Coats and colleagues reported that Cambodian mother-infant dyads in Prey Veng province had significantly lower whole blood thiamine diphosphate concentrations, regardless of infant clinical beriberi diagnosis, compared to American controls [5]. Lower eThDP were reported in a representative survey of Cambodian women of childbearing age residing in urban Phnom Penh and rural Prey Veng provinces compared to a small convenience sample of purportedly thiamine-replete Canadian women from Vancouver [25,26].

Unfortunately there is currently a lack of consensus on the most appropriate cut-offs to define suboptimal status or deficiency using whole blood ThDP or eThDP, and there are no nationally representative data available on biochemical thiamine status of any population group in Southeast Asia. Therefore, the objective of this study was to determine eThDP among women of childbearing age and their children aged 6–69 mo who participated in the most recent 2014 Cambodian Demographic and Health Survey (CDHS) [27] and the linked National Micronutrient Survey [28] to determine the prevalence of thiamine deficiency in this population using various cut-offs.

## Methods

### Study design and data collection

This biochemical thiamine analysis was part of the 2014 Cambodian National Micronutrient Survey [28] (conducted June 2 –December 12, 2014), which was linked to the CDHS, a nationally-representative survey of adults aged 15–49 y and children from 24 Cambodian provinces [27]. Population proportionate to size sampling was used to select 611 villages from which 16,356 individual households were selected. Trained, Khmer-speaking enumerators visited all selected households and collected information on health outcomes including nutrition, fertility and family planning, morbidity and mortality, housing, and assets and wealth using a validated, standardized questionnaire [27]. One week to 2 months after the CDHS had visited the household, one sixth of the households were re-visited and biological samples were collected from mothers and their children. Full sampling and survey details can be found elsewhere [27,28]. With one sixth of the households re-visited, it was estimated that 935 mothers and children could be included in the micronutrient survey, but due to absence of mother or care-takers and refusal to participate, blood samples were collected from only 726 women and 781 children, and eThDP was measured in 726 and 761 samples, respectively. Pregnant women (*n =* 7) were excluded, leaving 719 maternal samples for analysis. Hemoglobin concentrations were measured by the CDHS survey, and not repeated during the micronutrient survey. As hemoglobin was measured in only half of the children and women, data on hemoglobin and anemia prevalence is only available for 441 women and 476 children.

### Ethical approval

The National Ethics Committee for Health Research, Phnom Penh, Cambodia granted ethical approval (057 NECHR 2014) for the 2014 Cambodian National Micronutrient Survey. Eligibility for participation included: having participated in the CDHS and have given permission to the CDHS team to be re-visited for the micronutrient survey, a child in the household aged 6–59 mo, neither mother nor child having evidence of severe or chronic illness, and mothers or care-taker providing written, informed consent. All data was anonymized.

### Blood collection

Nurses collected non-fasting, morning-time blood samples into trace element-free, heparin-coated tubes (Vacuette, Greiner Bio One, Austria) at a central village location. Samples were stored in a dark cooler box and transported to the nearest Provincial Health Centre within 6 h of collection. Samples were centrifuged (3000 g for 10 min), plasma and buffy coat were thoroughly removed, and erythrocytes were separated into 500 μL aliquots and frozen at -20°C. Frozen samples were then transported to the Department of Fisheries Post-Harvest Technologies and Quality Control Laboratory, Fisheries Administration in Phnom Penh for storage at -20°C. Once the survey was completed the samples were batch shipped on dry ice to Abbot Laboratories in Singapore for eThDP analysis.

### Laboratory analysis

After arrival of the blood samples in Abbott Nutrition R&D Singapore Center, the samples were stored at -78°C. eThDP was measured using a modified method of Lu & Frank [29], as reported elsewhere [30]. Briefly, samples were thawed on ice in a dark room with amber light. Trichloroacetic acid solution was added to precipitate protein out. After centrifugation, the supernatant was collected, washed with methyl tert-butyl ether, and subject to ultra-high performance liquid chromatography (Agilent model 1290 system, Singapore) with a fluorescence detector (Agilent model G1321A, Agilent Technologies, Singapore) and an autosampler (Agilent model G4226A, Agilent Technologies, Singapore) that allowed online pre-column derivatization with potassium ferricyanide.

### Thiamine status cut-offs

At least ten different thiamine status cut-offs are reported in literature for use in women and children [5,21,22,31–40]. Noteworthy are the Institute of Medicine (IOM) definitions of thiamine deficiency (eThDP <70 nmol/L) and marginal deficiency (70–90 nmol/L) [22], which are based on values from 68 healthy Dutch blood donors and laboratory staff aged 20–50 y [31]. In the original citation these are described as cut-offs for whole blood or red cells [41], causing confusion over the correct biological sample for their use. If the IOM cut-offs represent whole blood ThDP, then values should be corrected for hematocrit to obtain eThDP values [34]. For example, the Coats *et al*. noted that their lab uses a reference range of 80–150 nmol/L for whole blood ThDP, or, if divided by hematocrit, 150–290 nmol/L for eThDP [5]. The Institute of Medicine cut-offs have not been employed here due to confusion surrounding use for whole blood versus erythrocyte ThDP.

We have made a distinction between cut-offs being reported as falling below a ‘reference range’, or ‘deficient’ or ‘marginally deficient’, as it is unclear whether a value outside a reference range represents real deficiency. For example, a thiamine deficiency cut-off of eThDP <180 nmol/L was proposed by Mancinelli and colleagues to align with the 25^th^ percentile eThDP of 103 healthy controls (45 men and 58 women, employees of University “La Sapienza” Hospital) in Rome, however none of these subjects were thiamine deficient [38]. In best practice, an average of 120 subjects are needed to generate accurate reference limits for a given biomarker [42]; this has not been the case for the majority of thiamine cut-offs.

We present four cut-off values describing suboptimal status below a reference range: the abovementioned eThDP <180 nmol/L [38]; eThDP <165 nmol/L, the lower bound of a 95% reference range (165–286 nmol/L) of 48 (25 men and 23 women) healthy hospital staff at Broadgreen Hospital, Liverpool, UK [32]; <150 nmol/L, corresponding to the eThDP reference range of the Mayo Medical Laboratories [5]; and <140 nmol/L, the lower limit of normal eThDP (cut-off of lowest 2.5%) of healthy blood donors in Christchurch, New Zealand; *n* unknown [33]. eThDP <148 nmol/L was used to categorize low thiamine status in two studies [35,36], and originated as the lower bound of normal range (50–150 ng/mL packed cells) from 21 healthy adults in Nashville, Tennessee [37]. Since the values of 148 and 150 nmol/L are close, we have used only <150 nmol/L as a cut-off in the current paper.

Marginal thiamine deficiency has been described using one cut-off, eThDP between 120–150 nmol/L, a cut-off reported in [21,39], but no details of these values are known. Two cut-offs for thiamine deficiency have been reported: eThDP <120 nmol/L, again that was used in [21,39], but the origins of this cut-off are unknown; and <118.5 nmol/L, which was used to categorize thiamine deficiency in [35], and is described as below the 95 percentile reference range (40–85 μg/L) among healthy black South African adults in [40]. As these values are close again, we have used <120 nmol/L as cut-off for thiamine deficiency.

### Data analyses

Demographic characteristics were computed as mean (SD) or n (%), and eThDP as mean (95% CI). Children’s eThDP and thiamine status are categorized by age category, 6–12 mo, 13–24 mo, 25–36 mo, 37–59 mo, and ≥ 60 mo. A t-test was performed to compare women and children’s eThDP, and eThDP among residents in rural and urban areas; a one-way ANOVA was employed to compare eThDP among different wealth quintiles, and children’s age categories (with least significant difference post-hoc correction for multiple comparisons).

Linear regression models were built to measure the association between eThDP and various independent variables. Variables were included in the linear regression model if *P* < 0.20 in bivariate correlation, and were entered stepwise into separate models for women and children. The following variables were evaluated for inclusion in the model for both women and children: province, wealth quintile, population density (urban/rural), mother’s education, cigarette smoke in home, subsidized healthcare available for household, weight, height/length, age, and hemoglobin concentration. The model for women also included BMI, and the children’s model also included sex and birth order.

Results were considered significant at *P* < 0.05. Weight-for-age, height-for-age, BMI-for-age, and weight-for-height z-scores were calculated using WHO Anthro and WHO Anthro Plus software programs, otherwise all analyses were performed using SPSS for Macintosh version 23.0 (IBM, Armonk, NY, USA).

## Results

Demographic characteristics are shown in **Table 1**. Women were 30 (6) y, and the majority had a normal BMI (70%; 18.5–24.99 kg/m^2^), were married (93%), and had attended some formal schooling (93%). Children were 41 (17) mo and 46% were girls; 28% (*n =* 130) of children were wasted (weight-for-age z score < -2 SD), and 39% (*n =* 182) were stunted (height-for-age z score < -2 SD).

**Table 1:** Demographic characteristics of Cambodian women (16–49 y) and children (6–69 mo).

Characteristic	n	Mean (95% CI) or n (%)
**Women**		
Age, *y*	719	30 ± 6
BMI, *kg/m*^*2*^	450	22.0 ± 3.9
*Underweight (≤ 18*.*5)*		57 (13%)
*Normal (18*.*51–24*.*99)*		313 (70%)
*Overweight (25–29*.*99)*		9 (14%)
*Obese (≥ 30)*		15 (3%)
Hb, *g/dL*	440	12.0 ± 1.3
*Anemia*, *Hb <12*.*0 g/dL*^a^		186 (42%)
Marital status	719	
*Married*		669 (93%)
*Divorced/separated*		28 (4%)
*Widowed*		18 (~2%)
*Single*		4 (~1%)
Schooling attended	719	
*None*		119 (17%)
*Primary (1–6 y)*		385 (~54%)
*Lower Secondary (7–9 y)*		162 (~23%)
*Upper Secondary (10–12 y)*		53 (7%)
**Children**		
Sex, *n (%) girls*	761	348 (46%)
Age, *mo*	761	41 ± 17
Hb, *g/dL*	469	10.9 ± 1.2
*Anemia*, *Hb <11*.*0 g/dL*^a^		230 (30%)
Weight-for-Age z-score	472	-1.45 ± 1.03
*Weight-for-Age < -2 SD*		130 (28%)
*Weight-for-Age < -3 SD*		23 (5%)
Height-for-Age z-score^b^	472	-1.61 ± 1.22
*Height-for-Age < -2 SD*		182 (39%)
*Height-for-Age < -3 SD*		45 (10%)
Weight-for-Height z-score^c^	399	-0.74 ± 0.98
*Weight-for-Height < -2 SD*		29 (7%)
*Weight-for-Height < -3 SD*		6 (2%)
BMI-for-Age z-score	472	-0.60 ± 1.03
*BMI-for-Age < -2 SD*		28 (6%)
*BMI-for-Age < -3 SD*		10 (2%)
**Household**		
Exposure to cigarette smoke at homestead	719	
*Daily*		376 (52%)
*Weekly*		59 (8%)
*Less frequently*		48 (7%)
*Never*		236 (33%)
Receive subsidized healthcare	719	
*Free healthcare*		110 (15%)
*Subsidized healthcare*		33 (5%)
*Not subsidized*		576 (80%)
Homestead toilet facility	719	
*Flush toilet*		372 (52%)
*Rudimentary latrine*		19 (3%)
*No facility/bush defecation*		328 (45%)

^a^ Anemia cut-offs using unadjusted hemoglobin concentration (g/dL) from [43]

^b^ *n* = 1 BMI-for-age Z score and *n* = 3 height-for-age Z scores were identified extreme outliers and removed from analysis.

^c^ weight-for-height z score was computed only for children aged 6–60 mo (*n =* 399), as it is undefined for children >60 mo

Children had a higher mean (95% CI) eThDP of 174 nmol/L (171–179 nmol/L) compared to women, 150 nmol/L (146–153 nmol/L; *P* < 0.001); **Table 2**. eThDP did not differ between children living in rural (173 nmol/L, 169–177 nmol/L) versus urban areas (180 nmol/L, 172–189 nmol/L; *P =* 0.14), however, rural women had lower eThDP (146 nmol/L, 143–150 nmol/L) compared to their urban peers (164 nmol/L, 157–171 nmol/L; *P* <0.001). Compared to higher wealth quintiles, eThDP was lower among both children (*P* = 0.04) and women (*P* < 0.001) in lower wealth quintiles. Young children aged 6–12 mo had significantly lower eThDP (144 nmol/L, 130–159 nmol/L) compared to older children aged 13–36 mo (176 nmol/L, 170–173 nmol/L; *P <* 0.001) and > 36 mo (177 nmol/L, 172–182 nmol/L; *P <* 0.001); eThDP in the latter two age groups did not differ (*P* = 0.90).

**Table 2:** eThDP of Cambodian women (16–49 y) and children (6–69 mo).

	Women (16–49 y)			Children (6–69 mo)		
	n	eThDP (nmol/L)		n	eThDP (nmol/L)	
		Mean (95% CI)	Range		Mean (95% CI)	Range
All	719	150 (146–153)	41–352	761	174 (171–178)	66–379
Residence						
*Urban*	145	164 (157–171)	82–301	151	180 (172–189)	73–339
*Rural*	574	146 (143–150)	41–352	610	173 (169–177)	66–379
Wealth quintile						
*Lowest*	159	136 (128–143)	43–273	183	165 (157–173)	66–327
*Second*	152	142 (135–148)	41–281	171	172 (164–179)	82–340
*Middle*	128	155 (145–164)	68–352	135	178 (168–188)	73–342
*Fourth*	129	153 (146–160)	52–272	148	179 (171–187)	78–311
*Highest*	151	167 (159–175)	66–325	124	183 (173–193)	66–379
Age						
*6–12 mo*	-	-	-	50	144 (130–159)	66–281
*13–24 mo*	-	-	-	108	171 (161–182)	83–323
*25–36 mo*	-	-	-	154	180 (171–188)	80–340
*37–59 mo*	-	-	-	325	176 (170–182)	71–379
*≥ 60 mo*	-	-	-	124	178 (169–186)	85–218

**Table 3**shows the percentage of women and children below selected cut-offs. Using the most conservative cut-off for thiamine deficiency (eThDP < 120 nmol/L), 27% of mothers and 15% of children were thiamine deficient. Worrisome, 38% of infants were thiamine deficient.

**Table 3:** Thiamine status (eThDP, nmol/L) of Cambodian women of childbearing age (15–49 y) and children (6–69 mo) using various published cut-offs.

Thiamine status cut-offs	Women	Children					
	*n =* 719	All	6–12 mo	13–24 mo	25–36 mo	37–59 mo	≥ 60 mo
		*n =* 761	*n =* 50	*n =* 108	*n =* 154	*n =* 325	*n =* 124
**Lower than reference range**							
< 180 nmol/L [38]^a^	558 (78%)	441 (58%)	35 (70%)	66 (61%)	89 (58%)	183 (56%)	68 (55%)
< 165 nmol/L [32]^b^	481 (67%)	347 (46%)	33 (66%)	53 (49%)	70 (46%)	138 (43%)	53 (43%)
< 150 nmol/L [5]^c^	398 (55%)	271 (36%)	29 (58%)	43 (40%)	47 (31%)	111 (34%)	41 (33%)
< 140 nmol/L [33]^d^	336 (47%)	217 (29%)	26 (52%)	36 (33%)	38 (25%)	86 (27%)	31 (25%)
< 135 nmol/L [34]^e^	292 (41%)	188 (25%)	25 (50%)	31 (29%)	30 (20%)	77 (24%)	25 (20%)
**Marginal thiamine deficiency**							
120–150 nmol/L [21,39]^f^	208 (29%)	158 (21%)	10 (20%)	24 (22%)	32 (21%)	62 (19%)	30 (24%)
**Thiamine deficiency**							
< 120 nmol/L [21,39]^f^	192 (27%)	114 (15%)	19 (38%)	19 (18%)	15 (10%)	49 (15%)	12 (10%)

^a^ 25^th^ percentile of *n =* 103 (45 men and 58 women) healthy controls, employees of University “La Sapienza” Hospital, Rome, Italy [38]

^b^ lower bound of 95% reference range (165–286 nmol/L) of *n =* 48 (25 men and 23 women) healthy hospital staff at Broadgreen Hospital, Liverpool, UK [32]

^c^ reference range of 80–150 nmol/L for whole blood ThDP, or 150–290 nmol/L for eThDP equivalent (ThDP divided by hematocrit) [5]; note that eThDP < 148 nmol/L was used to categorize low thiamine status [35,36] from the lower bound of normal range (50–150 ng/mL packed cells) from *n =* 21 healthy adults [37]

^d ^lower limit of normal eThDP (cut-off of lowest 2.5%) of healthy blood donors in Christchurch, New Zealand; *n* unknown [33].

^e ^eThDP reference range of 135–330 nmol/L among *n =* 33 healthy Italian volunteers (18–50 y) [34]

^f^ cut-off reported in [21,39]; details unknown; note that eThDP < 118.5 nmol/L was used to categorize thiamine deficiency in [35]; described as below the 95 percentile reference range (40–85 μg/L) among healthy black South African adults in [40]

The following variables were included in the eThDP prediction linear regression models: for children, age (mo), hemoglobin concentration (g/dL), and household wealth quintile; for women, household wealth quintile, household qualification for subsidized health, province of residence, population density (rural/urban), education level attended, and hemoglobin concentration (g/dL) were included. Hemoglobin concentration was the only predictor of children’s eThDP (adjusted R^2^ = 0.024, standardized β [95% CI], 0.161 [3.2–11.3 g/dL], *P* < 0.001). The model for women’s eThDP (adjusted R^2^ = 0.044) included wealth quintile (standardized β [95% CI], 0.209 [3.6–10.0], *P* < 0.001) and hemoglobin concentration (standardized β [95% CI], 0.114 [0.6–7.9 g/dL], *P* = 0.02).

## Discussion

Here we present the first nationally representative biochemical thiamine status data from a country in Southeast Asia, a region where beriberi still exists [5–7]. Despite variation in the prevalence of thiamine deficiency by cut-off, there is clear evidence of suboptimal thiamine status among women of childbearing age and their children in Cambodia. Of highest concern are infants aged 6–12 mo (*n* = 50), of whom 38% was classified as thiamine deficient by the most conservative cut-off, and up to 70% using the most liberal one (eThDP < 180 nmol/L). Although not included in this study, Cambodian infants aged 0–6 mo, who are at the highest risk for developing infantile beriberi [6,8] due to the relatively high thiamine needs compared to their body size [8], are likely to have a poor thiamine status too. Given the peak in infant mortality around 3 months of age in Cambodia, combined with the biochemical evidence of a high prevalence of thiamine deficiency in the population, we are convinced that infant beriberi is a highly under recognized cause of death in Cambodia, and that infants <12 mo of age are at the highest risk for thiamine deficiency. Indeed, Kauffman *et al*. estimated that infantile beriberi might be responsible for 6% of overall infant mortality in Cambodia [44]. Whereas other causes of infant mortality have been addressed, leading to a considerable decrease in infant mortality over the last 2 decades [27], the peak at 3 months of age has remained (see Fig 1).

Children had significantly higher eThDP than their mothers (174 versus 150 nmol/L; *P* < 0.001). This is consistent with a recent study in Cambodia in which we measured eThDP among women (18–45 y) and their children (6–59 mo) in Prey Veng province as part of a randomized controlled trial investigating thiamine-fortified fish sauce [45]. Women and children in the control group (who received only nutrition education; 92 mothers and 87 children) had mean (95% CI) eThDP of 184 nmol/L (169–198 nmol/L) and 213 nmol/L (202–224 nmol/L), respectively. Coats and colleagues reported similar values among Cambodian women of childbearing age: 141 and 150 nmol/L (thiamine diphosphate in whole blood, corrected for hematocrit) among mothers of infants with and without the clinical symptoms of infantile beriberi, respectively [5]. The fact that Coats *et al*. reported ThDP concentrations in infants with beriberi and without beriberi that are close or within to the lower reference ranges for ThDP (<140 and <150 nmol/L [5,33]) suggests that these cut-offs might be too conservative. Alternatively, perhaps eThDP (or whole blood ThDP) is not a good predicator of beriberi. However, reports of beriberi are not uncommon in Cambodia and throughout Southeast Asia [5–7,18,19,46,47], suggesting that thiamine deficiency is an issue. Therefore our biochemical data suggesting a high prevalence of thiamine deficiency suggest that current cut-offs can indicate populations at risk for thiamine deficiency even though the cut-offs might not be clinically meaningful.

These Cambodian values do differ greatly from older, previously reported values among older children and adults in Europe. The mean eThDP of British adolescent girls and boys (*n =* 54, 13–14 y) was 226.8 nmol/L and 206.1 nmol/L, respectively [35], which are similar to those reported for free-living British elderly women (247 nmol/L, *n* = 80) and men (218 nmol/L, *n =* 57) [36]. However, due to advances in HPLC equipment and sensitivity since these latter studies were published two decades ago [35,36,39], there is merit in investigating whether different thiamine deficiency cut-offs need to be developed for adults and children.

As shown in Table 3, there is wide variation in the prevalence of thiamine deficiency and/or suboptimal thiamine status depending on the cut-off employed, therefore it is difficult to determine the severity of low thiamine status as a public health concern in Cambodia and the wider region. However, even with the most conservative cut-offs, >25% of the mothers, and 38% of the infants were classified as deficient in our study, making thiamine deficiency a serious public health concern, which is also reflected in the peak in infant mortality around 3 months of age. Wilkinson *et al*. used the lowest 2.5% of healthy blood donors in Christchurch, New Zealand to set their deficiency cut-off of eThDP < 140 nmol/L, but astutely noted that “the lower limit of normal, seen in a healthy population, cannot be assumed to be the upper limit of abnormal” [33]. While the cut-offs shown in Table 3 may be helpful in categorizing potentially at-risk individuals, it is clear that more research is required to develop clinically meaningful thiamine deficiency cut-offs. Although a recent review has not found compelling human nutrition trials to prioritize an update of recommended dietary thiamine intakes [48], this may be due to a heavy focus on beriberi as an outcome. Thiamine is involved in important cell functions including glucose conversion and energy metabolism in the Kreb’s cycle and pentose phosphate pathway [49]. Thiamine also performs critical enzymatic functions in processes related to brain development and function, neuronal communication, as well as immune system activation, signaling and maintenance [50]. There is evidence that obesity may impair thiamine utilization and alter requirements: a recent American study reported thiamine deficiency in 15.5–29% of obese patients seeking bariatric surgery [51]. In addition, considerable evidence over the past century has linked thiamine deficiency to neurological problems including cognitive deficits and encephalopathy [52]. Even a short-term exposure to poor thiamine intake in early life may have long-term impacts on cognition [53,54]. It is difficult to establish new thiamine cut-offs without new human studies on these manifestations of thiamine deficiency that are distinctly different from beriberi. Perhaps a wider description of clinical syndromes of thiamine deficiency is needed, compiling all under one term, for example, Thiamine Deficiency Disorders, just as advancing insights into iodine deficiency in the early 1980s led to the use of the term Iodine Deficiency Disorders. And perhaps circulating thiamine concentrations might not sufficiently reflect thiamine status. We further urge future researchers to collect beriberi prevalence data from clinical settings with matched biochemical samples to better guide development of cut-offs that have clinical and/or physiological meaning.

This study has several strengths, but most notably it is the first nationally representative evaluation of eThDP in a country in Southeast Asia (while a national study in the Philippines using erythrocyte transkelotase activity is the most recent in the region in two decades [55]). Thiamine status was measured among women of childbearing age and children because, due to unequal household food distribution and higher needs relative to body size, these groups are at highest risk of nutritional deficiencies in low-income countries. These are also the most pertinent population groups for biochemical thiamine assessment because thiamine-deficient mothers confer a higher risk of infantile beriberi [9–11], and in turn mortality [6], to their children. There is evidence that increased thiamine intake improves biochemical thiamine status among mothers and their infants in Cambodia [30]. During a recent randomized controlled trial in Prey Veng we found that maternal consumption of thiamine-fortified fish sauce over 6 mo throughout pregnancy and lactation resulted in higher eThDP among mothers and infants, as well as breast milk thiamine, compared to a control sauce containing no thiamine [30], indicating that this group shows potential for improvement in eThDP. However, thiamine deficiency may be common among the elderly [56], and beriberi outbreaks among adult men have also been reported [47,57], likely due to higher thiamine needs with increased physical activity and a high-carbohydrate diet [22,49,58]. Therefore future studies should include the full range of population groups.

Dietary intake data was not collected in this study, therefore while it is well established that low dietary thiamine intake causes low biochemical thiamine status [22], and that there is little thiamine available in the Cambodian diet [15,59], we cannot provide direct causation for low eThDP in this population.

Consistent with recent studies of thiamine status in Cambodia, we report low eThDP among a nationally representative sample of Cambodian women and their young children. Thiamine status classification varies dramatically depending on the cut-off employed, from 27% to 78%, and 15% to 58% among mothers and children, respectively. More research is required to develop more useful, clinically meaningful thiamine status cut-offs. However, in view of the peak in infant mortality around 3 months of age suggestive of infantile beriberi, immediate action is required to develop interventions to increase thiamine intake in Cambodia and the wider Southeast Asia region where thiamine deficiency and beriberi remain a public health concern.

## Supporting information

S1 File Strobe checklist. (DOC)
